# Book Review

**Published:** 1900-09

**Authors:** 


**An Epitome of the History of Medicine.** By ROSWELL PARK, M.D. Second Edition. Pp. xiv., 370. Philadelphia: The F. A. Davis Company. 1899.—In a notice of the former edition of this work we expressed the indebtedness of the profession to Dr. Roswell Park for the useful and compendious History of Medicine which he had published. The necessity for a second edition within a year shows how general the appreciation of his work has been. Some inaccuracies which appeared in the previous edition have been corrected—one which was pointed out in our review has been substantially, though not completely, rectified. The book has been enlarged by an additional chapter, on what the author calls iatrotheurgic symbolism, in which he displays a tendency to discover a phallic import in things symbolical. In this supplement he strangely misnames the celebrated leader of the Argonauts. We would again commend this excellent treatise.

**How Surgery became a Profession in London.** By D'ARCY POWER. Pp. 55. London: The Medical Magazine Association. 1899.—In these pages, reprinted from a series of articles in the *Medical Magazine*, we can trace the development of the modern surgeon from the two early types of "the military surgeon and his peaceful—though often brawling—brother the barber-surgeon" of the middle ages. It forms an interesting chapter of medical history, which may be read with pleasure and profit by those who are interested in the origin of the surgical craft.

**Lord Lister and Surgery.** By ROBERT TURNER, M.D. Pp. 28. London: Henry J. Glaisher. 1899.—It is well that we should be reminded from time to time of the great work done by Lister, which is liable to be forgotten amid the constant introduction of new details of wound-treatment, and this pamphlet deals in an easy and elementary fashion with the revolution introduced by that great master and reminds us of our indebtedness to him.

**Ludwig and Modern Physiology.** By J. BURDON-SANDERSON. Pp. 365–379. Washington: Government Printing Office. 1898.—This pamphlet, from the Smithsonian Report for 1896, is divided into three portions: (1) an introduction, (2) Ludwig as investigator and teacher, and (3) the old and the new vitalism. The first part deals with the methods and scope of physiological enquiry, and touches on the relation between adaptation and the struggle for existence, and the birth of modern physiology under the parentage of Ludwig. The second is an eloquent appreciation of the great and many-sided physiologist, whose long life was devoted to a successful attempt to place the study of function on a truly scientific basis. The third and concluding part of the pamphlet is a somewhat controversial consideration of the different schools of vitalists and non-vitalists, amongst the former of whom Professor Burdon-Sanderson ranges himself and Ludwig. The subject is treated in that careful and thoughtful manner so characteristic of the author, and is, moreover, a most interesting character sketch of Professor Ludwig.

**Recent Advances in Science, and their bearing on Medicine and Surgery.** By MICHAEL FOSTER. Pp. 339–364. Washington: Government Printing Office. 1898.—This pamphlet from the Smithsonian Report for 1896 is a reprint, from *Nature*, of the first Huxley lecture delivered at Charing Cross Medical School. It contains a fine account of the state of sciences cognate to medicine at the time when Huxley was a student, and of the great steps forward in physiology which have been made since then. The lecture abounds in pregnant thoughts—for instance: "Each case of illness is to the doctor in charge a scientific problem, to be solved by scientific methods." "The value of a discovery is to be measured not only by its immediate application, theoretical and practical, but also by the worth of the idea it embodies and to which it gives life." "An attitude of mind once gained is a possession for ever, far more precious than facts which are gathered in with toil and flee away with ease." It is a worthy "preface," as Professor Foster calls it, to the Huxley lectures.

**Recent Progress in Physiology.** By MICHAEL FOSTER. Pp. 437-452. Washington: Government Printing Office. 1898.—Rather late in the day we have received the reprint of the President's address to the physiological section of the British Association for the Advancement of Science, delivered at Toronto in 1897. The period considered comprises the years which separate 1884 and 1897, or the two meetings of the Association in Canada. The Professor deplores the strong "commercial" element which is appearing in even so uncommercial a science as physiology; but finds comfort in three marked features of progress. The first is the *new* physiological chemistry, which seems to him to have most happily combined the two sciences, physiology and chemistry,—the one not trying to produce a chemistry of physiology, nor the other a physiology of chemistry. The second feature is the increasing attention given to the study of the lower forms of life, whereby we make it the scaffolding on which to build the more complicated explanations of vertebrate life. The third advance is in the line of what we call "internal secretion." The address ends with a tribute to the magnificent work of Golgi, and his method of studying the central nervous system, in which it is carefully pointed out that Golgi, in introducing the *technique*, at the same time pointed out the theoretical significance of the result produced by the method, with which are coupled the names of Ramon y Cajal and Kölliker. The address is worth reading, if only for noticing the masterly style of the author.

**A Pocket Medical Dictionary.** By George M. Gould, M.D. Fourth Edition. Pp. 9—837. London: H. K. Lewis. 1900.—Although we have been for many years well acquainted with Dr. Gould's invaluable lexicographical work, this *multum in parvo*, already in its fourth edition, comes to us as a novelty. It is printed on such appropriate paper, that, notwithstanding the many pages, its description as a "pocket" volume is quite justified. Its tables of bacteria, muscles, nerves, *râles*, and eponymic terms give it a special claim upon our attention, and when we state that it provides not only such a simple definition as "blind, without sight," but also tells us what meta-amidophenylparamethcxyquinolin is, it will be seen that its range is large. Dr. Gould informs us that "old" is "advanced in life"; but he has not ventured on a definition of "young," although we learn that a "maid" is "a young unmarried woman." This dictionary allows us to use "chemical" as an alternative to "chemic," which is evidently Dr. Gould's favourite; but his conservatism does not permit "surgic" instead of "surgical." If we did not feel sure that "continueter" for "let it be continued" is a misprint, we should begin to think that Dr. Gould was going to lay his reforming hand on the Latin as well as the English tongue.

**The Processes of Life Revealed by the Microscope: a Plea for Physiological Histology.** By Simon Henry Gage. Pp. 381—396. Washington: Government Printing Office. 1898.—This pamphlet, the address of the president of the American Microscopical Society, reprinted from the Smithsonian Report, is a sketch of the life-history of cells as revealed by modern research, and of the changes in cells which accompany their various activities, such as rest and movement, secretion, and so on. It is a plea for the study of cellular structures from a physiological, as distinguished from a morphological, point of view.

**Some Aspects of the Mental Discipline Associated with the Study of Medicine.** By C. O. Hawthorne, M.B. Pp. 48. London: J. & A. Churchill. 1899.—This thoughtful address, delivered to a body of ladies belonging to the Queen Margaret College Medical Club, may be perused with profit by all engaged in the practice of medicine. As the title implies, it deals with the habit of mind produced by the investigation of disease, and the unconscious mental training which is thereby received. The author shows in a very interesting manner the education in the appreciation of evidence or testimony which the examination of patients gives: he quite rightly points out that success in treatment largely depends on the power which the doctor acquires of sifting the evidence presented to him, of acting upon what is essential, and rejecting the irrelevant. Further, a medical man who has learnt his lesson knows that he should not express an opinion without having duly considered the evidence. The author gives credit to the medical world for intellectual candour and freedom from prejudice: he thinks that the practice of medicine, far from blunting, stimulates the imagination, whilst at the same time it makes a man value accuracy of statement. On these and other grounds he contends that the discipline of medicine is in itself a broad and liberal education.

**Golden Rules of Medical Practice.** By ARTHUR HENRY EVANS, M.D. Pp. 71. Bristol: John Wright & Co. [1899.]—No exception can be taken to most of the condensed aphorisms herein contained. To those who already know more, it is possible that the little book in the pocket might sometimes serve as a useful reminder of what should be in the head; but to those who do not know, a concentrated essence of this kind must be comparatively as useless as the "ox in the teacup." The size of the book is such that if not in the pocket, it is not likely to be found elsewhere.

**Lectures on Clinical Medicine: delivered in the Glasgow Royal Infirmary.** By JOHN LINDSAY STEVEN, M.D. Pp. viii., 195. Glasgow: Alex. Macdougall. 1900.—Clinical lectures on rare cases, such as Landry's paralysis, scleroderma with hemiatrophy, and osteo-arthropathy, are always of interest, inasmuch as by this teaching of the lecturer, he becomes instructive to the reader or the listener. Such selections from the case-book of a physician, become worthy of the attention of a wider circle than the usual class-room of a hospital, where it has now become the fashion to decry clinical lectures in favour of more desultory work in the wards. The author has given us a well printed and well illustrated volume, easy to read, and instructive on every page.

**Tuberculosis: its Nature, Prevention, and Treatment, with special reference to the Open Air Treatment of Phthisis.** By ALFRED HILLIER, M.D. Pp. xii., 243. London: Cassell and Company, Limited. 1900.—The author believes "that a concise manual, dealing with all the hydra heads of Tuberculosis in one volume, will form a work of reference of some interest and value to practitioners of medicine and medical students." It is for them that this work is primarily intended. One of the most important points in the whole book relates to the control of the sputum (p. 127). "The absolute control of the sputum of the consumptive must be gradually insisted on, and the use of the pocket spittoon, and other precautionary measures, become a routine practice with a phthisical patient, whether he be in a sanatorium, a lodging-house, a private dwelling, or anywhere else." The author does not appear to encourage the practice of burning the sputum direct with the paper spitting-cup; the practice of collecting the sputum in carbolic acid is inferior to the burning method. The following paragraph summarises the greater portion of the book, and therefore all the modern views on phthisis: "It will be tolerably clear, from what has already been written, that consumptives of both sexes, and all classes, should recognise that they are called upon to entirely adapt their lives, for the sake of themselves, their families, and their neighbours, to the requirements of their disease. . . . It is highly desirable that the open-air treatment should be adopted. The best introduction to this which a patient can possibly have is a residence of some months in a sanatorium conducted on open-air principles, and it is to be hoped that ere long these will be available for every class of the community. The benefit derived from a stay in such an establishment is not merely the improved health of the patient; it is largely the education, which teaches him that some of the measures and precautions most desirable for his own welfare are also an essential part of his duty to his neighbour." We appear as yet to be only on the threshold of the sanatorium question.

**Consumption: its Nature and Treatment.** By William H. Spencer, M.D. Pp. 83. London: Henry J. Glaisher. 1900.—"This is no compilation. What I tell of, that have I seen; what I teach, that has experience taught me." The book is based upon lectures to Bristol students, but of a previous generation, inasmuch as two Professors of Medicine have followed the author since his resignation: it is now intended to instruct the intelligent and educated public. This is done "in choice and scholarly, albeit somewhat old-fashioned diction." (*The British Physician*, April 16th, 1900, p. 275.) The author's method of treatment, by what he calls the "Diffusion" method, depends mainly on medicated inhalations by means of the Arema vaporiser. This is diametrically in antagonism to the more modern and popular views on open-air treatment: the two are absolutely incompatible with each other. Both have advocates; time alone can decide which is the more efficient.

**Nordrach at Home; or, Hygienic Treatment of Consumption Adapted to English Home Life.** By Jos. J. S. Lucas. Pp. 60. Bristol: J. W. Arrowsmith. [n.d.]—This useful little book shows what those patients who are not able to leave home can do for themselves there. The author does not think that hygienic treatment can be so well carried out in private houses, away from the personal influence and discipline of the director; still, in many cases much may be done by means of a few simple directions such as he gives. It is generally admitted that the system of treatment is far and away more important than the climate. Food, fresh air, and rest are the chief agencies required.

**The Schott Methods of the Treatment of Chronic Diseases of the Heart.** By W. Bezly Thorne, M.D. Third Edition. Pp. 132. London: J. & A. Churchill. 1899.—The third edition of this book contains a few additional cases, some of which are interesting and of the nature one would expect to obtain benefit from the Nauheim treatment. There are people past middle life who experience pain and cardiac distress started by over-exertion. Possibly the circulatory disturbance in such cases is not due simply to cardiac weakness. The vaso-motor system may be largely at fault. The tepid baths and carefully regulated exercises restore the normal balance.

**The Murmurs of Mitral Disease.** By Edward Mansfield Brockbank, M.D. Pp. viii., 47. Edinburgh: Young J. Pentland. 1899.—Dr. Brockbank argues in support of the theory that the so-called presystolic murmur of mitral stenosis is not presystolic but systolic. It must be confessed that a strong case is made out in favour of the systolic theory. Perhaps one of the strongest points advanced is in connection with the crescendo character of the murmur. Dr. Brockbank explains the rise of pitch and the short first sound which frequently follows the end of the murmur by the ventricular systole driving blood through the narrowed and stiffened orifice, the edges of which are approaching under pressure and ultimately come into sharp contact. Although we consider that Dr. Brockbank states his case in an able manner, we do not think he has proved his contention.

**Notes on Malaria in Connection with Meteorological Conditions at Sierra Leone.** By Major E. M. Wilson. Third Year. Pp. 15. London: H. K. Lewis. 1899.—We have on a previous occasion reviewed Major Wilson's pamphlets on malaria and expressed our favourable opinion of the views held. This year's copy contains some new notes on 1898. There is no direct reference to the most recent work on mosquitoes and malaria.

**Pye's Surgical Handicraft.** Fourth Edition. Revised and edited by Bertram M. H. Rogers, M.D. Pp. xvi., 550. Bristol: John Wright & Co. 1900.—We are glad to see another edition of this useful little book, of deserving popularity amongst house-surgeons. Our first impressions were favourable. The volume is no larger than it was fifteen years ago, yet most modern processes and apparatus have been introduced, including a chapter on skiagraphy. The book has many points to recommend it, but on careful perusal we much regret to find many antiquated or erroneous teachings. Particularly is this so in dealing with that important subject, hemorrhage. Severe hemorrhage from vessels of all kinds is to be treated by plugging (p. 3); severe bleeding from an aneurysm of the femoral requires first a tourniquet, and then plugging and pressure (p. 45); secondary hemorrhage is generally arrested by plugging and compression (p. 47). We should be sorry for any house-surgeon who made such blunders. Davy's dangerous lever (p. 13), puncture of the bladder *per rectum* (p. 445), and triangular bandages should, in our opinion, have been left out. The treatment of cut throat is not up to date. Hallex (p. 311) should read hallux.

**Aseptic Surgery.** By CHARLES BARRETT LOCKWOOD. Second Edition. Pp. xiv., 264. Edinburgh: Young J. Pentland. 1899.—The present edition of this useful little book contains "no alterations of any moment" from the first edition, which we have previously reviewed. The author still uses the same methods of obtaining asepsis as before. He regards the biniodide of mercury as the most efficient antiseptic, and claims that with it he has had better results in the disinfection of the skin and that wounds have done better. We are pleased to say that this accords with our own experience, and can heartily commend the book to students and practitioners. "Binioeide" appears in the headline of p. 143.

**The Student's Handbook of the Surgery of the Alimentary Canal.** By A. ERNEST MAYLARD, M.B., B.S. Pp. xv., 510. London: J. & A. Churchill. 1900.—The present work is an abridged and amended edition of the author's treatise on the same subject, which we have previously reviewed. The abridgment has been mainly effected by the elimination of the detailed reports of cases which appeared in the larger volume, and this we regard as an improvement. The author has done his work well, and the book contains all that was worth knowing in the previous edition without its needless encumbrances.

**Intestinal Obstruction.** By FREDERICK TREVES. and·New Revised Edition. Pp. xi., 565. London: Cassell and Company, Limited. 1899.—The first edition of this work, published in 1884, filled a distinct want in the literature of intestinal surgery. The present issue further embodies the extensive additions which have been made to our knowledge of the subject during the past fifteen years. Intestinal obstruction is one of the bugbears of abdominal surgery, and the operation-mortality of such cases has made many surgeons act as if the best thing to do is to avoid any surgical interference as long as possible. Mr. Treves's forcible teaching is just the reverse of this. In clearly expressed sentences he points out that in acute intestinal obstruction, abdominal taxis, puncture of the intestines, and other methods of treatment are for the most part feeble excuses for delay and for avoiding operation in which the mortality increases the longer it is delayed. There is a multiplicity of causes and varieties of causes of intestinal obstruction which render the subject difficult to handle; but it has been dealt with by a master hand, and the book must take foremost rank in its branch of abdominal surgery. The book is divided into three parts—pathology and morbid anatomy, clinical manifestations, and treatment. Half of the work is taken up with the pathology and morbid anatomy, which might with advantage have been curtailed. We think it would have been better to deal with acute obstruction as a whole first, and subsequently to take up chronic obstruction. That there are difficulties in arrangement we have already admitted; but in reading the book the impression is given that the morbid anatomy, clinical symptoms, and treatment of any form of intestinal obstruction are unnecessarily dissociated. We heartily commend this work, which is destined to become classical.

**Diseases of the Gall-Bladder and Bile-Ducts, including Gall-stones.** By A. W. Mayo Robson, Assisted by Farquhar Macrae, M.B. Second Edition. Pp. 313. London: Baillière, Tindall and Cox. 1900.—The arrangement of the present is an improvement on that of the former edition, in which the Hunterian lectures for 1897 remained in their original form. An index, too, has been added, which is essential for reference. It is doubtful whether the inclusion of the tables should be continued in the next edition; a summary of the results (which have not been excelled by anyone) would be sufficient. We know of no better work on the subject, and the author's wide experience and successful results have brought the surgery of this region to a high level of excellence.

**Contributions of Original Articles to Orthopedic Surgery.** By James W. Cokenower, M.D. Pp. 32. Des Moines: Conaway & Shaw. 1898.—The commonplaces of this branch of surgery are to be found in this series of isolated papers. Though they may have had the interest of locality for the societies before which they were read, there seems no other reason for collecting them into a pamphlet. The author has nothing very new to say, and his ground is mostly safe from controversy. On the subject of osteoclasis, however, he rather leaves this safe position; but his chief advocacy of it, as opposed to osteotomy, seems to be on the score of its freedom from sepsis. Perhaps it is unnecessary to add that Dr. Cokenower has his own modification of the Grattan osteoclast.

**A Manual of Gynæcological Practice.** By Dr. A. Dührssen. Second English Edition, translated and edited from the Sixth German Edition by John W. Taylor and Frederick Edge, M.D. Pp. xlii., 279. London: H. K. Lewis. 1900.—We have previously reviewed the first edition of this work. The present edition contains such alterations as became necessary by the lapse of time. The teachings of its pages are thoroughly sound, but the subject is too briefly treated. The illustrations as a whole are poor.

**Notes of Fifty Consecutive Cases of Cœliotomy for Diseases of the Uterus and Appendages, with Forty-eight Recoveries, performed within the last twenty months, with observations.** By James Macpherson Lawrie, M.D. Pp. 56. London: John Bale, Sons & Danielsson, Ltd. 1899.—Nearly half the cases here recorded consisted of removal of the appendages—an operation of low mortality. Of the four abdominal hysterectomies for uterine fibroid one died. The book consists almost entirely of an enumeration of cases in which the details given are frequently too brief to allow of comment.

**Gynæcological Nursing.** By G. A. Hawkins-Ambler. Pp. viii., 96. London: The Scientific Press, Limited. 1900.—This is a sound little book, and should be found useful by the nurses for whom it is written. We should like to have seen some reference to the administration of enemata by means of a tube and funnel.

**Aids to the Diagnosis and Treatment of Diseases of Children (Medical).** By John McCaw, M.D. Second Edition. Pp. 241. London: Baillière, Tindall & Cox. 1899.—The call for a second edition of this work has enabled the author to include a few omissions of his first issue, and we think by this the book has been increased in value. Students will find Dr. McCaw's "Aids" of use in getting a general outline of children's diseases, but for greater detail recourse must be made to some of the larger works on this subject. We have yet to learn what degree of eminence warrants an author being quoted without a prefix of Dr. or Mr. There is a delightful irregularity in this work on this point, but we refrain from quoting names lest those without the prefix should be puffed up with pride thereby.

**De la Trachéo-Thyrotomie dans le Cancer du Larynx.** Par le Dr. E. J. Moure. Pp. 20. Bordeaux: Feret et Fils. 1898.—Dr. Moure here records four cases of thyrotomy for cancer of the larynx, and speaks highly of the results of this operation. He gives some good directions for the carrying out of the operation, which has been so successful in his hands.

**The Hygiene of the Mouth.** By R. DENISON PEDLEY. Pp. 93. London: J. P. Segg & Co. [N.D.] —This is a well-written treatise, with several good and serviceable illustrations. The author has simplified his work by arranging it all under two chapters: The Hygiene of the Mouth in Children; and The Hygiene of the Mouth in Adults. The chemical theory of caries of the teeth is here well emphasised. Yet it must be borne in mind that teeth of faulty structure are more readily attacked than those that are well and properly developed. As regards caries, the only cure is its early removal from the teeth; the cavity thus made should be filled with gold or other filling material. This, of course, means periodical visits to the dental surgeon; because if a patient waits for pain in a tooth before giving attention to it, the caries will by that time have penetrated the pulp, and if this has happened the pulp must be either capped or killed, and the nerve canals and pulp cavity disinfected and filled. Children should be taught to use their teeth by biting crusts and hard food. To give a child soft food only is "as wise as he who would ride *always* in a carriage in order to develop his legs." Mr. Pedley also judiciously deplores the drinking of water at meals, and thereby failing to mix the food with saliva before swallowing. He points out that decayed teeth often originate other diseases. His remarks on the cleansing of the mouth are very important; although the rules he lays down are rather stringent, and it is doubtful whether the constant and vigorous use of the tooth-brush, powder, and tooth-pick is not liable to do more harm than good to teeth of faulty development. Of course, there are some teeth to which the author's rules would be very beneficial; but these are exceptional. The author refers to necrotic teeth, by which we understand him to mean teeth which have lost their pulps and nerves and the periosteal membrane; those which have lost their pulps and nerves only, and remaining tight, are called dead teeth. It is possible to save dead teeth by treatment, even if they are causing abscess; but if they are necrosed, that is, having lost their source of nourishment from outside as well as inside, they must be extracted. If persons would carry out Mr. Pedley's advice we believe those distressing pains so often endured would be very much lessened or altogether prevented.

**Pyorrhœa Alveolaris.** By JOHN FITZGERALD, L.D.S. Pp. 62. London: The Medical Publishing Company, Limited. [1899.]—This is a *résumé* of what is known on the subject—carefully collated no doubt, but only fairly well written. In the main the treatment advocated is practical, but there are suggestions which appear purely theoretical and which are scarcely to be considered feasible or satisfactory. Take, for instance, replantation as a method of treatment.

**Materia Medica and Therapeutics.** By J. MITCHELL BRUCE, M.D. [Fifth Edition]. Pp. xii., 609. London: Cassell and Company, Limited. 1899.—It is superfluous to express an encomium upon Dr. Mitchell Bruce's well-known text-book. This edition has been revised, considerably enlarged, and in all ways brought up to date in respect both of the pharmaceutical and therapeutical sections. It is especially to be commended that the author does not confine himself strictly to those substances which are made official to the British Pharmacopœia, but includes also some other useful and important articles. We would most strenuously recommend this book to our students, and beg them to study it. The existing regulations of the Conjoint Board tend to encourage in students an ignorance of remedies and therapeutics which is simply deplorable.

**The Newer Remedies.** By VIRGIL COBLENTZ, Ph.D. Third Edition. Pp. 147. Philadelphia: P. Blakiston's Son & Co. 1899.—This book contains a great deal of information not to be found in the text-books. The author, as Professor of Chemistry in the New York College of Pharmacy, has vast opportunities for research, and his experience as a teacher enables him to present a list as complete as possible of many new drugs, on which it would be difficult otherwise to obtain any authentic information. It is a formidable list, including many proprietary drugs in daily use, as well as many other preparations which are not likely to be of much service.

**Rough Notes on Remedies.** By WM. MURRAY, M.D. Third Edition. Pp. x., 142. London: H. K. Lewis. 1899.—The additions to this issue are chapters on Specific Disease, Ptyalism in Jaundice, Turpentine Vapour in the Treatment of Pneumonia, and Liqueur Brandy. The author remarks that "what we most need is a close acquaintance with the virtues and potentialities of our remedies so that we may be ready to use them whenever a rare or unusual crisis presents itself." Liqueur brandy is doubtless a very efficient form of alcohol and ethers.

**The Pocket Pharmacopœia.** By FREDERICK HUDSON-COX and JOHN STOKES, M.D. Pp. 206. London: Baillière, Tindall and Cox. 1899.—Following the plan of the former edition of 1885, written by the late Dr. Armand Semple, the writers have endeavoured to make the book more generally useful as a medium for acquiring a knowledge of the materials and therapeutics of the British Pharmacopœia. By the elimination of about 150 pages of appendices and index, and by using very small type, they have produced a pocket edition of 206 pages instead of the 535 of the Pharmacopœia. Readers of and above middle age will prefer the larger publication.

**Le Mont-Dore et ses Eaux minérales.** Par le Docteur Em. Emond. Quatrième édition. Pp. 252. Paris: J.-B. Baillière et Fils. 1900.—This new edition of a well-known guide to the waters of Mont-Dore has somewhat grown, inasmuch as it contains the further results of a long-continued experience of twenty-five years' study and observation on the spot. The author directs especial attention to his views on phthisis, asthma, pharyngitis, laryngitis, and coryza.

**Xeroform.** Pp. 88. London: Burgoyne, Burbidges & Compy. [n.d.]—This *brochure* contains various papers setting forth the value of xeroform as an antiseptic. Xeroform or tribromophenol-bismuth is a fine yellow powder of neutral reaction, with a slight phenol-like odour, tasteless, and unaffected by light. It is said to be superior to iodoform in that it is non-poisonous, practically odourless, never causes eczema, is free from germs, has a powerful action upon bacteria and is sterilizable by simple heating.

**Shaw's Manual of the Vaccination Law.** By a Barrister-at-Law. Seventh Edition. Pp. xv., 256. London: Shaw & Sons. 1899.—Although the sixth edition of this work was issued immediately after the publication of the order of 1898, another edition has been required. It will be found of service to those charged with the carrying out of the Act, which came into operation January 1st, 1899. This Act, like most others, has imperfections and shortcomings, which will, it is to be hoped, receive attention at a future date. But there can be no doubt that the immediate result of carrying vaccination to the homes of children in England has considerably increased the number of those who have been submitted to this wholesome discipline during the past year.

**Aids to the Analysis of Food and Drugs.** By T. H. Pearmain and C. G. Moor. Second Edition. Pp. 206. London: Baillière, Tindall & Cox. [1899].—In the production of this edition many of the more important sections, especially that on milk, have been re-written and extended with advantage; but as the processes formerly given for the assay of medicinal preparations are now omitted, reference being made simply to the methods of the new Pharmacopœia, the work is but slightly increased in bulk, and still correctly claims to be the handiest compendium of information relating to the analysis of foods.

**Buxton: its History, Waters, Climate, Scenery, &c.** Pp. 36. Manchester: Henry Blacklock & Co. Ltd. [n.d.]—This booklet gives all needful information about a place whose attractions cannot fail to be recognised, without undue puffing. The book consists largely of advertisements, and is, of course, itself one.

**Creuznach-Spa and its Environs.** Pp. 62. Wiesbaden: Petmecky Brothers. 1899.—This little book, well written and well illustrated, not beset by advertisements, but constituting a good general and medical guide of the pretty health-resort of the valley of the middle Rhine, is quite a model of what a good guide book to a spa should be.

**Eastbourne as a Health Resort.** By CHARLES ROBERTS. Pp. 31. London: Bale, Sons & Danielsson, Ltd. 1899.—Book-making becomes easy when a magazine article is reprinted and issued in this form. We are told that Eastbourne differs from most of the towns on the south coast of England in having been laid out from the first as a health resort; and, further, that a stationary marine hospital would give all the supposed advantages to be derived from sea voyages. We are surprised to learn that "gastric complaints in old people do well in spite of, and possibly in consequence of, the hardness of the water"; whereas in other towns the hardness of the water is usually considered to be an objectionable feature. Doubtless we shall soon hear of a sanatorium for the systematic treatment of phthisis in this favoured spot.

**The Natural Waters of Harrogate.** By FRANCIS WILLIAM SMITH, M.D. Pp. 101. London: Dawbarn and Ward, Limited. 1899.—This book is dedicated to "the doctors of this country for the good of their patients and the glory of Harrogate." The waters are "chemically, therapeutically, and clinically considered, with reference to their application by drinking and bathing; by the light of fresh analysis, and by examination of the blood." The essence of the latter point is comprised in the following sentence: "When hæmoglobin is higher than corpuscles, it indicates a gouty and apoplectic tendency; when the reverse is the case, anæmia is indicated."

**Golden Rules of Psychiatry.** By JAS. SHAW, M.D. Pp. 74. Bristol: John Wright & Co. [1899.]—We recommend this little book to the general practitioner. Certain sections of it would prove useful even where no previous knowledge of mental disease existed; but presuming a certain acquaintanceship with this subject to be possessed by most medical men, the author has made his epitome cover a good range of clinical matter. The work is clearly and concisely written, and will, no doubt, fulfil a need; the section on examination and certification of an insane person is especially serviceable.

**The Power of Nature in Disease.** By J. WALLACE ANDERSON, M.D. Pp. 50. Edinburgh: William F. Clay. 1899.—Dr.

Anderson gives us a learned though inconclusive essay on the power of nature in healing disease. It is divided into two parts. The first is a sketch of the history of the doctrine from the time of Hippocrates downwards. The second part discusses the ways of nature in dealing with various diseases—*e.g.*, fever, hemorrhage. We may perhaps take the former subject as an instance of the thesis defended. The author alleges "that the state of fever generally is the expression or evidence of nature's endeavour to overcome and expel the disease." We must confess that we do not believe such a doctrine, which appears to us to imply that the tissues act with a purpose—an assumption which involves, at bottom, a foreseeing consciousness on the part of the cells of which our bodies are built up. Modern science is more inclined to represent fever as simply a reaction to influences, whether microbic or not, acting on the body, with no ulterior end in view. Whatever view one takes, however, this little book is well worth reading both as a record of the views of a long succession of our forefathers in the art of medicine, and as an ingenious defence of such tenets.

**A Guide to the Examinations by the Conjoint Examining Board in England, and for the Diploma of Fellow of the Royal College of Surgeons of England.** By FREDERICK JAMES GANT. Seventh Edition. Revised by WILLMOTT EVANS, M.D. Pp. 252. London: Baillière, Tindall and Cox. 1899.—A careful reading of this book shows that it is a useful digest of the rules and regulations of the Conjoint Board, together with the papers set at the examinations during the last eight years, with examples of the *vivâ voce* ordeals undergone by the candidates. This evidently has supplied a want, or else it would not have reached its seventh edition; it reminds us of the talk of a students' room, but it is free from the exaggeration of the disappointed and the slights of the successful.

**Experimental Proofs of the Role of Alcohol.** By E. MACDOWEL COSGRAVE, M.D. Pp. 39. Dublin: Fannin & Co. 1899.—"Alcohol is not a stimulant, it wears us out, but does not increase our work. *Short life, less work, worse work*—that is the output of alcohol. What does it give us in exchange? It offers to narcotise our minds as well as our bodies, to hide from us the truth about ourselves; to make us satisfied when we ought to be ashamed; and to substitute dreams for realities." This is Dr. Cosgrave's summary of his observations. We have heard it all before. His facts are not new, and we are unconvinced by his statements.

**Schoolboys' Special Immorality.** By MAURICE C. HIME, LL.D. Second Edition. Pp. 48. London: J. & A. Churchill. 1899.—The vice of masturbation is often, one may truly say generally, begun in ignorance during the earlier years of boyhood, under the influence of some physical defect or from bad example, but no schoolmaster can be held guiltless of culpable negligence if he ignores the all too frequent existence of the habit and takes no precautions against it. Because it is always a disagreeable matter to approach, a false sense of delicacy has led many kindly and even thoughtful masters—and we must add parents —to withhold the timely warning and admonition that would in most cases check this unnatural nastiness. We therefore cordially welcome this small *brochure*, which offers discreet and much-needed advice to both parents and schoolmasters in clean, straightforward, and acceptable language.

**The Medical Annual.** Bristol: John Wright & Co. 1900.—This most popular work has reached its eighteenth year of publication, and fully maintains its useful character. It gives, in a condensed form, a large amount of information, partly obtained from current literature and partly in the form of original articles by well-known writers, thus presenting the reader with a good summary of modern advances in medical knowledge. One of the most valuable articles is by Major Ronald Ross, giving an account of his brilliant discoveries respecting the nature and prevention of malarial fever, and giving also, in a simple form, a good outline of the work of others on this important subject. Professor Loomis of New York writes on the subject of phthisis, devoting attention chiefly to treatment. He remarks that one point in treatment which is steadily gaining ground is that the destruction of the bacilli and the neutralisation of their products, so far unsuccessfully attempted from the outside by germicides and antitoxins, may be accomplished from within by the living tissues. Hygienic treatment consists in strengthening the organism to effect its own cure. Mr. Hurry Fenwick writes on "Disorders of the Prostate Gland," and naturally devotes attention to Bottini's operation, which has so much come into vogue of late. No less than forty-eight pages are devoted to "Diseases of the Stomach," Dr. Boardman Reed of Philadelphia writing on the medical aspects and Mr. Walter G. Spencer on the surgical. Both of these writers have done their work with much ability. Among local contributors, Mr. F. Richardson Cross writes on "Ophthalmology" and Dr. P. Watson Williams on "Differential Diagnosis of Nasal Accessory Sinusitis." The volume should undoubtedly find a place in the library of every practitioner.

**Transactions of the Clinical Society of London.** Vol. XXXII. London: Longmans, Green, & Co. 1899.—In this volume we have the usual varied and interesting cases with which these transactions always abound, bearing witness to the large store of clinical material brought before the society, and to the indefatigable energy of its members. There are numerous well-executed skiagrams, coloured and other plates illustrative of the text.

**Transactions of the Royal Academy of Medicine in Ireland.** Vol. XVII. Dublin: Fannin and Co. 1899.—This volume contains the usual reports and lists of officers, members, and fellows. There are sixteen papers in the section of medicine, eleven in surgery, seven in obstetrics, twenty-two in pathology, seven in state medicine, and three in anatomy and physiology. It would be invidious to select one or more papers when all are of a high standard and express the views of the heads of the profession in Ireland. An excellent series of plates shows the œdema and muscular atrophy resulting from beri-beri.

**Transactions of the Iowa State Medical Society.** Vol. XVII. Waterloo, Iowa: H. G. Middleditch. 1899.—This is the report of the transactions of the forty-eighth annual congress of the society. The papers and the discussions on them are all interesting reading, perhaps one of the best being the paper and discussion on the "Serum Treatment of Diphtheria." Again we notice the proof-reading wants improving, for we came across such words as "ager," "asexnalization," and "tempature."

**Transactions of the Michigan State Medical Society for the Year 1899.** Vol. XXIII. Grand Rapids: Published by the Society. 1899.—To judge from the papers and discussions in this volume, very good work is done by the Michigan State Medical Society at its Annual Meeting. Amongst the papers we would draw attention to especially are, one by F. W. Robbins, on "Urethral Disease and Marriage," and the one entitled "Why Some Severe Cases of Appendicitis Recover Without Operation," by J. W. Carstens. The discussions on the papers are apparently reported verbatim, which we are inclined to think is a mistake. A condensed account of the remarks would add very much to the dignity of the reports of the proceedings.

**Transactions of the Medical Society of the State of New York.** Published by the Society. 1899.—The great variety of matters considered in this volume makes it impossible, without running to a length we cannot afford, to adequately review this contribution to modern medicine. Of the forty-nine articles many will repay careful consideration, for they are the products of the minds of some of the leading practitioners of the State of New York. It seems a pity that so much good work should not have a larger circulation in this country.

**Transactions of the Ohio State Medical Society.** Cleveland: Press of J. B. Savage. 1899.—During its fifty-fourth meeting the society appears to have got through no inconsiderable amount of work, and had good discussions on some of the papers. We suggest to the publication committee to head the pages with the titles of the articles, as readers find it more convenient.

**Transactions of the College of Physicians of Philadelphia.** Third Series. Vol. XX. Philadelphia: Printed for the College. 1898.—Some miscellaneous papers of the usual excellent type are to be found in this volume. Dr. Deaver pleads for prompt interference with cases of typhoid perforation: he states that "the occurrence of sudden acute abdominal pain, with very decided abdominal rigidity and tenderness, with or without collapse, is in a typhoid-fever patient the strongest possible indication for immediate abdominal section. To wait after the advent of these symptoms for further corroborative evidence of perforation is fatal." Drs. H. A. Hare and C. A. Holder give an interesting estimate of the Brand bath in typhoid fever, and ask the question whether the routine use of the bath is wise, answering it by saying that "a rigid routine is unadvisable." Dr. Arthur V. Meigs gives reasons why the Philadelphia Board of Health practice of placarding houses in which are persons suffering with scarlet fever and other contagious diseases should not be continued. The method of placarding does not appear to have been of much service, and in various ways has been productive of harm. A paper on the abuse of ergot in the treatment of hemorrhage gives the views of Dr. Frederick A. Packard, as follows: "In treating medical hemorrhage our object should be to endeavor to favor the formation of a clot in the ruptured vessel by measures that increase the coagulability of the blood, by calcium chloride, or, where possible, by local applications (such as the topical use of such remedies as hamamelis in epistaxis, the gentle inhalation of turpentine or other hæmostatic vapor in hæmoptysis, the administration of tannic acid in hæmatemesis, of acetate of lead in hemorrhage from the bowel), to prevent mechanical disturbance of the clot by producing local rest (opium to check cough and to stop peristalsis), and to use our best efforts to lessen blood-pressure (as by restriction of fluid ingestion, by the use of saline laxatives when permissible, by the hot foot-bath, by ligature of the extremities, by veratrum viride, nitroglycerin, or venesection in various classes of cases), but, above all, to avoid any cause for increase in blood-tension, and especially to abstain from the use of ergot, which is, above all other drugs, the most active in lessening the capacity of the arterial tree." This appears to us to be a very terse and excellent summary of what to do and what to avoid in the treatment of hemorrhage.

**Transactions of the Grant College Medical Society, Bombay, 1898.** Bombay: "Tattva-Vivechaka" Press. 1899.—The President has the candour to admit that the members of the society did not take interest in it, and that there had been no substantial work done during the past year; nevertheless this number contains much interesting and useful information on the utility of Haffkine's inoculations as prophylactic against the plague.

**Transactions of the Dermatological Society of Great Britain and Ireland.** Vol. IV. London: H. K. Lewis. 1898.—No doubt there are reasons, and perhaps good and only temporary ones, for the smallness of this volume; but it is not a healthy sign. The clinical work brought forward is, however, upon the whole good. Dr. P. Abraham has two good plates of xanthoma diabeticorum; Dr. A. Eddowes, a plate of ringworm contracted from a pet hedgehog; Mr. W. Anderson illustrates a case of an obscure form of erythema induratum; and Dr. Savill gives the details of a very exceptional skin manifestation, probably caused by angeio-neurotic disturbance, and which is interestingly demonstrated by picture-drawings of the complaint on the left wrist and hand.

**Transactions of the American Laryngological Association.** New York: D. Appleton and Company. 1900.—The work of this very representative gathering of American specialists is as valuable as any of its predecessors. To the general physician the series of four original contributions on the relation of pathological conditions of the ethmoid region to asthma will be especially interesting, although we cannot believe that the nose is quite so important a causal factor of asthma as some of the writers in this volume do.

**Transactions of the American Association of Obstetricians and Gynecologists.** Vols. IX., X., XI. Philadelphia: Wm. J. Dornan. 1896–8.—There is much in these volumes which will interest the abdominal surgeon as well as the gynæcologist, and the contributors are amongst the foremost of American practitioners. Nearly every subject of gynæcological interest is discussed, and discussed ably, in these volumes, which have several very good full-page plates. Volume X. contains a memorial article on Mr. Greig Smith, "edited" by Dr. L. S. McMurtry, from the *Bristol Medico-Chirurgical Journal*, and illustrated by an excellent portrait of our late distinguished colleague.

**Transactions of the American Ophthalmological Society.** Vol. VIII. Part 2. Hartford: Published by the Society. 1898.—The report of the committee on ophthalmia neonatorum deserves special mention. It states that of about 50,000 blind persons in the United States more than 5,000 are blind from ophthalmia neonatorum; and that if the 2 per cent. nitrate of silver method was universally adopted (as advocated by Credé) the number of blind from this cause might in a single generation be reduced to

350 cases, or even less. The relative frequency of blindness in rural districts is entirely due to the neglect of this or some other similar precaution. Following the lead of the Ophthalmological Society of the United Kingdom, the American Society passed a resolution advocating the adoption of the Credé method, and urging its compulsory use in almshouses and lying-in institutions. Nearly one quarter of the volume is devoted to records of growths in or near the eye. Dr. E. Gruening relates a case of lipoma of the orbit. Probably this is the only undisputed case on record. Three cases of buphthalmia (hydrophthalmia) occurring in three sisters are reported by Dr. W. B. Johnson. In the youngest the disease was arrested by iridectomy. There are several other important articles, and amongst them is one by Dr. James Minor, bearing the somewhat sensational title, "Learning to See at Forty." The volume contains some excellent illustrations, chiefly from photographs, and some capital drawings from microscopic specimens.

**Transactions of the American Pediatric Society.** Vol. XI. 1899.—The present volume opens with Dr. W. P. Northrup's presidential address on "Methods of Instruction in Pediatrics." The address, with the discussion which followed, is of interest, as embodying the ideas of the leading American authorities on the subject, and also showing what a high position the teaching of pediatrics holds in the curriculum of the American medical student. Dr. Henry Koplik, in a paper on "Pulmonary Hemorrhage following Exploratory Puncture of the Chest for Fluid in Infants and Children," utters a word of warning against the indiscriminate use of the exploratory needle merely for purposes of more exact diagnosis. We feel that the warning, coming from one who has had such great clinical experience, may profitably be borne in mind, during the present wave of bacteriological enthusiasm. Dr. R. G. Freeman's preliminary observations "On the Separation of Bacteria from Milk by Natural Process" are of far-reaching importance, as they may be a very decided step in the direction of providing infants with raw milk free of germs. The remaining papers of the volume fully sustain the reputation of the Society, and we can recommend Dr. J. D. M. Miller's paper on "Acute Articular Rheumatism in Infants Under One Year," and Dr. Emmett Holt's on "The Importance of Prolonged Rest in Bed after Acute Cardiac Inflammations in Children," as worthy of careful perusal.

**Transactions of the American Surgical Association.** Vol. XVII. Philadelphia: William J. Dornan. 1899.—The treatment of appendicitis seems still to form the favourite topic of discussion among American surgeons. Three papers are found in the present volume dealing with this subject from different points of view, and all tending to show the thoroughness with which it has been studied on the other side of the Atlantic. A valuable paper on "Some Conditions of Healing by First Intention, with Special Reference to Disinfection of Hands," is contributed by Dr. Theodore Kocher, of Berne, and many other papers of great surgical interest make up a volume fully equal to its predecessors.

**Guy's Hospital Reports.** Vol. LIII. London: J. & A. Churchill. 1898.—The volume opens with a charming article entitled "Notes on Diagnosis" by Dr. Pye-Smith, who has lately bidden adieu to active work at Guy's, and who writes with all that scholarly grace for which he is noted. Dr. B. A. Richmond and Dr. Alfred Slater contribute an important practical paper on "The Ætiological Significance of the Diphtheria Bacillus and its Variants," in which they claim to demonstrate that neither (1) the morphological character, nor (2) the virulence, nor (3) the toxin-forming power of a diphtheria bacillus, bears any constant relation to the clinical nature of the case from which it has been isolated; and that all diphtheria-like bacilli (including the pseudo or Hofmann organism) are really only varieties of the same species, because even the non-virulent forms can by appropriate treatment be converted into actively pathogenic and typical Klebs-Löffler bacilli. It is evident that the whole subject of the relations between diphtheria and the different varieties of bacilli needs much further investigation. Other interesting articles in this volume are furnished by Dr. Brice Poole on "An Account of some Cases occurring in the Maidstone Typhoid Fever Epidemic," and by Mr. Hastings Gilford on "The Surgical Treatment of (Unperforated) Gastric Ulcer, with an Account of Three Cases in which Operations were Performed."

**King's College Hospital Reports.** Vol. V. London: Adlard and Son. 1899.—This volume is very similar to those which have preceded it. The list of subscribers is extending, and the editors are well satisfied with the reception which has been given to the previous volumes. Part IV. of Dr. Curnow's historical sketch reminds the reviewer that of the staff of thirty years ago, the times of Fergusson and Partridge, very few are now surviving.

**The Middlesex Hospital. Reports of the Medical, Surgical, and Pathological Registrars for the Year 1897.** London: H. K. Lewis. 1898.—Accuracy and detail characterise these reports, which record, as usual, a large number of surgical renal cases. We note special tables and abstracts of cases of hernia, appendicitis, renal affections, diphtheria, enteric fever, and of surgical gynæcology, besides a complete resumé of pathological observations. The volume comprises nearly 400 pages, representing a vast amount of work by the registrars which can be emulated by many but imitated by few. We observe that twelve out of forty-nine renal operations are returned as "negative exploration of kidney,"—a rather large proportion in view of modern improvements in diagnostic measures, particularly considering that eight out of the twelve patients were females.

**Saint Bartholomew's Hospital Reports.** Vol. XXXIV. London: Smith, Elder, & Co. 1899.—Numerous papers of much interest and value are contained in this volume, and to all old Barts' men the reports will always have a very special interest, and will carry them back to days when they studied cases, not in its pages, but by the bedside of the patient. But many of the papers are of so much value, that the volume should be read by all interested in the progress of medical and surgical science. The first contribution to this volume is an exceedingly interesting case by Sir Thomas Smith—an abdominal cyst from which, after tapping, all food taken by the mouth, and all bile secreted, escaped. It was cured by pressure. Mr. Butlin's paper on the results of operations for cancer of the breast has been already referred to in the "Report on Surgery" in the June, 1899, number of this *Journal*. Mr. Butlin contributes another interesting paper, giving an account of his experiences of appendicitis at the hospital. We are glad to see his admission of the difficulty which often exists in the early stage of an acute attack in deciding as to whether there is general infection of the peritoneum. Dr. Walsham's paper on the X-rays in diseases of the chest will be of interest to physicians, but we must say we should be sorry to have to make the diagnosis from the reproductions of the skiagrams. The diagnosis of the complications of middle-ear disease is carefully considered in a paper by Dr. Armstrong Bowes. Mr. Marsh's contribution on "Points of Interest in Connection with Various Forms of Abscess" is of much practical value, and Mr. Walsham's experiences in operative surgery constitute a valuable record.

**Saint Bartholomew's Hospital Reports.** Vol. XXXV. London: Smith, Elder, & Co. 1900.—This volume of Saint Bartholomew's Hospital Reports contains much matter of practical utility. It is not often that obituary notices of two members of the staff have to be included in one volume, but such unfortunately is the case this year. The hospital sustained a great loss in the appointment of Dr. Kanthack, the pathologist and lecturer on pathology to the hospital, to the post of Professor of Pathology at Cambridge, and the Science of Medicine a yet greater loss by his early death. His connection with Saint Bartholomew's, and his enthusiasm for his work there, are described in a most interesting "In Memoriam" paper. Although only the initials of the writer are given, it is not possible to mistake those of Mr. Bowlby. The other obituary notice refers to a physician—Dr. Southey—who for many years had ceased to be connected with the hospital, but those of us who remember him during his visits to the wards will be glad to see this "In Memoriam" also. Mr. D'Arcy Power has given us his experience of operative surgery during the first sixteen months of his work as an assistant-surgeon, and he has certainly had many cases of interest during this period. Mr. Harrison Cripps also gives us tables of his abdominal operations. There are several valuable original papers dealing with some particular topic, which will be of interest both to the physician and surgeon, and an article descriptive of medical practice in foreign parts will interest many readers.

**Glasgow Hospital Reports.** Vol. I. Glasgow: James Maclehose and Sons. 1898.—We gather from the prefatory note that these Reports issue from the Royal, Western, and Victoria Infirmaries. The book contains many papers of much value. They deal with clinical observations and cases, and cover a wide range of subjects. To the student of clinical medicine and surgery such reports are invaluable. The first paper is by Professor M'Call Anderson on intra cranial tumours, and gives an account of six very interesting cases. A case of granuloma fungoides by Dr. McVail is an extraordinary one, and the patient, judging by the photographs, must have presented an appalling appearance. Three good examples of sudden blindness are reported by Dr. Maitland Ramsay, and then follows an able paper on the use of the ophthalmoscope in medical practice by Dr. James Hinshelwood. Dr. David Newman has an extremely valuable article on malformations of the kidney and displacements without mobility; he gives a very complete account of the subject, with references to the literature of the various conditions found; the cases are fully described, and illustrated by a number of well-executed pictures. Two other papers are on urinary asepsis, by Mr. J. H. Nicoll, and on the pathology of the coronary arteries of the heart, based on an analysis of 238 cases, with observations on the relation of disease of these arteries to angina pectoris and sudden death, by Dr. J. Lindsay Steven. There is also a useful paper on the clinical application of the Röntgen rays, by Mr. John Macintyre, and there are many other papers in the work which are of equal interest to those mentioned, but want of space prevents us from citing them by name. We desire to congratulate the committee and editors on the matter of their volume, which will repay careful reading, and also on its neat external appearance, the excellence of the type and illustrations, and last but not least on the provision of an index. The first of these reports promises well for the future.

**The Johns Hopkins Hospital Reports.** Vol. VII., Nos. 5-6-7-8-9. Baltimore: The Johns Hopkins Press. 1899.—The Reports of the Johns Hopkins Hospital are always of great value. This number is taken up entirely with a report on 459 cases of hernia, by Dr. J. C. Bloodgood, with special reference to cases operated upon by Halsted's method and transplantation of the rectus muscle, and is well worth careful attention. The details of his operation are very fully given and illustrated by a series of beautiful plates. Dr. Bloodgood has found an absence of the conjoined tendon in many cases of hernia, and in these cases he brings down the edge of the rectus muscle to prevent a recurrence of the hernia at this part of the inguinal canal. The proportion of cases in which suppuration has occurred has never been high in this series of cases, but since silver wire has been used instead of silk for sutures, and especially since rubber gloves have been worn by the operator and his assistants, the number of cases in which suppuration has occurred has been very small indeed. The small number of recurrences, too, has been very encouraging. The question of atrophy of the testis after excision of the veins of the cord, both in the radical cure of hernia and for varicocele, is exhaustively dealt with, and the groin operation for varicocele fully considered. The success of Trendelenburg's operation for varicose veins may induce surgeons to adopt the groin operation for varicocele.

**The Sanitary Value of the Operations of the Board in Reducing and Avoiding the Mortality from Typhoid Fever in the Metropolis of Sydney, N.S.W.**—The report of the medical adviser to the Board, Dr. Theo. Mailler Kendall, dated January, 1898, proves the triumph of advance in health due to progressive sanitation.

**Informes Rendidos por los Inspectores Sanitarios de Cuartel y los de los Distritos al Consejo Superior de Salubridad. Correspondientes al Año de 1898.** Mexico: Imprenta del Gobierno, en el ex-Arzobispado. 1899.—The pamphlet now before us was published a year after the improvements were made to which we called attention in December, 1899, and it records a substantial reduction in the number as well as in the mortality of zymotic diseases—notably in typhus fever, which was the scourge of the neighbourhood for more than 300 years. The medical men had considerable difficulty at first in convincing the inhabitants, and especially those of the poorer class, of the advantages of pure air, pure water, and cleanliness generally; but, after a time, they succeeded in inducing the people to work with them instead of against them, and the results so far have been in the highest degree encouraging.

**Burdett's Official Nursing Directory.** London: The Scientific Press, Limited. 1900.—This eminently useful directory contains much valuable information in addition to a list of trained nurses, with data as to their place and length of training (*e.g.*, a summary of the main facts regarding the various metropolitan and provincial training schools, an outline of the municipal laws affecting nurses, and some notes on provident funds available for nurses). It is a well-printed and very handy volume.

**Archives of the Roentgen Ray.** Vol. III. London: Rebman, Limited. 1899.—The interest and value apparent in the earlier volumes of the *Archives* are fully maintained in this, the third of the series. The general plan remains unaltered. The volume includes a number of papers bearing upon X-ray work and theory, some specially contributed, many abstracted from the proceedings of the Roentgen Society, as well as a large number of excellent half-tone plates reproducing skiagrams of special interest. Among much that will attract the attention of workers in this new field, Major Battersby's paper descriptive of his X-ray methods and achievements during the Soudan Campaign is calculated to take a leading place, not only as a chronicle of some of the first experiences in the systematic employment of such methods of diagnosis and localisation during warfare, but because of the valuable information of a more general kind that may be gathered from these experiences. Altogether, we may say that in these early days of skiagraphy, when every operator is but "feeling his way," such a record of actual successes and failures as the *Archives* affords is one which the practical worker cannot afford to ignore.

**The Edinburgh Medical Journal.** New Series. Vol. VI. Edinburgh: Young J. Pentland. 1899.—Most of the papers in this volume are of more than usual importance. The account of an outbreak of typhus fever, by Drs. Harvey Littlejohn and Claude B. Ker, demonstrates the value of unlimited fresh air, both for the success of the treatment of the patient and for ensuring the safety from infection of the attendants. We would also draw special attention to Dr. Beatson's paper on "Observations on the Existence of Enzymes in Cancerous Growths," in which he brings forward very strong arguments for their probable existence, although in the specimens he examined he was unable to demonstrate their presence. Papers by Mr. Barling on "Appendicitis," by Dr. Gibson on "Acromegaly," and by Dr. Kelynack on the "Pathology of Renal Tumours" are, amongst others, noteworthy. Local writers, we are glad to see, are well represented in the volume. The photographs illustrating some of the papers are much more distinct than usual, owing to the fact of their being printed on a white background. This is worth the attention of other journals.

**Annual Report [of New Medicinal Preparations] on the Year 1899.** Vol. VII. Darmstadt: E. Merck. 1900.—For those who wish to be amongst the advanced outposts in therapeutics this report is invaluable. It gives all the information possible on many drugs whose future reputation is likely to be in excess of present demands.

**The Humane Review.** April, 1900. London: Ernest Bell.—Our contemporary, we are informed in the introduction to this first number, has been founded to represent "the higher ethics

—the ethics of humaneness." It scarcely appears to have begun well in its leading article on "The Conflict between Science and Common Sense," by G. Bernard Shaw; but this view may be unjust, and due to the fact that the article in question is beyond our comprehension, being neither science nor common sense. Miss Honnor Morten writes a useful and sensible article on inhumanity in schools, and Mr. Josiah Oldfield has contributed a praiseworthy protest against the continuance of hanging as a punishment. We believe that *The Humane Review* will afford opportunities for the spread of much useful information, and hope that it will succeed in developing a higher sense of the obligations of humanity towards all living beings.

**On the Uses and Abuses of the Public Hospitals in Australia, Tasmania, and New Zealand.** By L. BRUCK. Pp. 72. Sydney: L. Bruck. 1899.—A perusal of this pamphlet shows clearly that the abuses of public hospitals at the antipodes are quite as serious as in the mother country, though the system of management is different. It will hardly be believed that in a progressive colony, such as we always considered New South Wales to be, there is no proper Medical Act, the stumbling block being the labour party—the representatives of the very people who benefit most by the work of the medical profession. Mr. Bruck is not a medical man; but evidently his sympathies are with the profession, and his small *brochure* is a heavy indictment of the manner in which medical institutions are created and managed.

**Index-Catalogue of the Library of the Surgeon-General's Office, United States Army.** Second Series. Vol. IV. D—Emulsions. Washington: Government Printing Office. 1899.—Now that the *Index Medicus* is, unfortunately, a thing of the past, the already great necessity of this work has become still further emphasised. The same careful compilation exists in the present volume as that which has characterised its predecessors. The last received includes 9628 author-titles, 8828 subject-titles, and 28,316 titles of articles in periodicals. No library can be considered complete without this catalogue, every page of which bespeaks a large amount of labour and success on the part of those engaged in its production.

**The Medical Digest, or Busy Practitioner's Vade-Mecum.** Appendix, including the years 1891 to March, 1899. By RICHARD NEALE, M.D. Pp. 261, xxxi. London: John Bale, Sons & Danielsson, Ltd. 1899.—The useful character of this work and the method of its compilation are so well known that they need little comment. The present volume has been incorporated with the appendix published in 1895, in order to facilitate reference. Dr. Neale is to be congratulated on the successful continuance of the valuable work, with which his name has so long been associated.

